# Photocatalytic-Driven Antiviral Activities of Heterostructured BiOCl_0.2_Br_0.8_ – BiOBr Semiconductors

**DOI:** 10.1021/acsomega.3c10310

**Published:** 2024-04-10

**Authors:** Razan Abbasi, Hani Gnayem, Yoel Sasson

**Affiliations:** Casali Center of Applied Chemistry, Institute of Chemistry, The Hebrew University of Jerusalem, Jerusalem 9190401, Israel

## Abstract

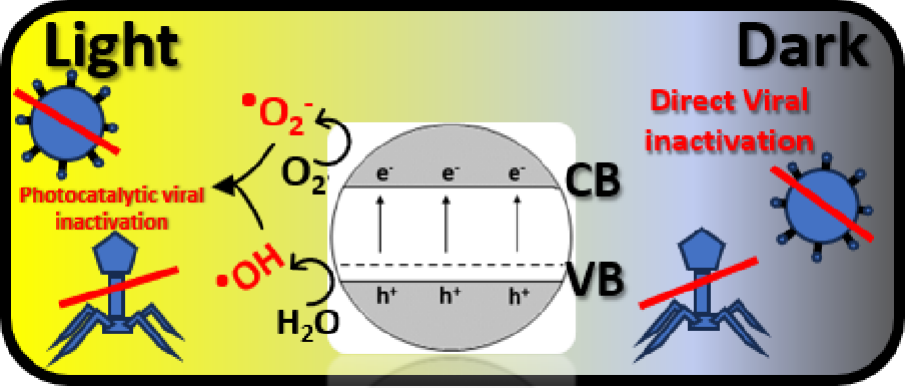

Numerous methods for eliminating severe acute respiratory syndrome coronavirus 2 (SARS-CoV-2) are being extensively examined in recent years as a result of the COVID-19 pandemic and its adverse effects on society. Photocatalysis is among the most encouraging solutions since it has the capacity to fully annihilate pathogens, surpassing conventional disinfecting methods. A heterostructured photocatalytic composite of (70%W BiOCl_0.2_Br_0.8_ with 30%W BiOBr) was prepared via a simple synthetic route that yielded microspheres ∼3–4 μm in diameter. The composite was evidenced to inactivate stubborn enveloped viruses. By utilizing scanning electron microscopy, transmission electron microscopy, N_2_ sorption, and X-ray diffraction, the morphology and the chemical composition of the heterostructured composite was revealed. Full elimination of SARS-CoV-2 occurred 5 min following the light-activation of the photocatalytic mixture. Illumination absence bared a slower yet effective result of full viral decomposition at a time span of 25 min. A comparable efficacious outcome was observed in the study case of vesicular stomatitis virus with complete diminishing within 30 min of visible light exposure.

## 1 Introduction

Numerous deaths are caused every year by infectious diseases owing to microorganisms, such as bacteria and viruses.^1^ Substantial awareness has been drawn toward this issue in the past few years, in particular since the spread of viral pandemics.^2^

The elementary structure of viruses comprises a genome of either DNA or RNA and a capsid, with additional features such as an extra protection layer of a protein-containing lipid bilayer envelope. Viral reproduction necessitates the utilization of the host’s cellular machinery, thus setting hurdles for drugs’ selective toxicity.^3^

The deleterious, alarming side effects of frequently used disinfecting methods, such as ozonation, chlorination, and UV radiation, intensified the requirement for more reliable approaches.^4^

Semiconductors are being widely explored as photocatalysts for their remarkable photoinduced capacity to inactivate infectious microorganisms,^5^ particularly severe acute respiratory syndrome coronavirus 2 (SARS-CoV-2) following the recent pandemic.^6^

Photocatalysis has the aptitude to diminish the viral spread without the inconvenient specificity entailed on medications.^7^ Antiviral photocatalytic activity was immensely explored, where Sjogren et al. showed the effective iron-aided titanium dioxide ability to disinfect phage MS2,^8^ Hu et al. tested Ag–AgI/Al_2_O_3_ activity against human rotavirus type 2, Wa,^9^ and Ditta et al. used copper oxide to eliminate Bacteriophage T4.^10^

The possible photocatalytic degradation mechanism of microbial cells by semiconductors was reviewed by Regmi et al.,^11^ where a couple of optional mechanisms were discussed. Starting with oxidative stress induction, i.e., the reactive oxygen species directly reacts with the microbial cell wall, oxidizes the outer membrane, and damages the genetic material. Another optional mechanism is the metal ion release, where the semiconductor releases metal ions that pass through the microbial outer membrane and interfere with the genetic material.

The third mechanism is a nonoxidative mechanism, reducing the critical cellular metabolism without the induction of oxidative stress.

The most interest is shown in the first mechanism, i.e., ROS generation. The prolonged ROS attack results in the oxidation of the viral matter, resulting in its deactivation and full oxidation to CO_2_ and water.

Recently, many semiconductors have been widely studied for their various beneficial photocatalytic applications in day-to-day life. Deciding their specific relevance depends on the unique properties of each semiconductor, including traits such as their size, reactivity, and crystal structure.^12^

Titanium dioxide, the prevalent and foremost photocatalyst, has a wide band gap tapering its activity to the UV region.^13^

ZnO–CdS composite was recently found to show biocidal activity under UV irradiation; however, CdS showed an increased cytotoxicity at high concentrations, limiting its biological applications.^14^

Photocatalytic activity via indoor light sources is crucial for banishing morbific microbes,^15^ thus bismuth oxyhalides are being extensively studied for their exceptional visible light-driven photocatalytic activities.^16−18^ They are already being implemented in an assortment of applications, namely pharmaceuticals^19^ and catalysis.^20^

Composite materials of bismuth oxyhalides, BiOX/BiOY, with X=Y=F, Cl, Br, or I, display an augmented visible light instigated action since they can accelerate the parting rate of photogenerated charge carriers, as well as a decrease in the recombination time.^21−23^

Xiao et al. synthesized BiOI/BiOCl composites with different ratios by a hydrothermal method in ethylene glycol using BiI_3_ and BiCl_3_ as precursors.

The composite of BiOI/BiOCl containing 10% BiOCl was found to have a high photodegradation rate constant of bisphenol A, specifically more than 4 and 20 times larger than those of pure BiOI and P25, respectively.^24^

The inimitable bismuth mixed oxyhalides family with the general structure of BiOCl_1–*x*_Br_*x*_, originally shared by our lab^25,26^ revealed higher photocatalytic capabilities opposed to BiOCl and BiOBr, separately.^27^

BiOBr has notable chemical stability and photoelectric characteristics.^28,29^ Yet, on its own, its rapid recombination of photogenerated carriers and low absorption efficiency in the visible light spectrum makes its photodegradation performance less adequate.^30,31^

Forming a heterojunction of BiOBr/BiOCl elevates its photocatalytic activity, since the layered structure of BiOCl comprises Cl^–^ plates enfolding [Bi_2_O_2_]^2+^ layers that produce an internal electrostatic field perpendicular to each layer, encouraging efficient parting of electron hole-pairs.^32−34^

Li et al.^35^ synthesized Bi_4_O_5_I_2_/BiOBr via in situ deposition-precipitation method and found it to have 3.43 times more photocatalytic activity against o-phenyl phenol compared to bare Bi_4_O_5_I_2_.^36^

Here, we propose a heterostructured visible light-activated photocatalytic composite prepared by the mechanical blending of BiOCl_0.2_Br_0.8_ with BiOBr. The dry yellowish powder exhibited extraordinary extermination proficiency of stubborn viruses, including the renowned pandemic-generating SARS-CoV-2 as well as vesicular stomatitis virus. Furthermore, the photocatalytic powder also acted against SARS-CoV-2 in the absence of illumination, broadening its applications range.

## 2 Methods

### 2.1 Materials

All materials were purchased from Sigma-Aldrich with a purity of ≥98% and were used without further purification.

### 2.2 Viral Strains and Culture Method

#### 2.2.1 SARS-CoV-2 Virus

Vero-E6 cells were grown in the DMEM medium supplied with Pen/Strep, glutamine, and 10% fetal calf serum. Twenty-four hours before the infection, cells were grown at 10^4^ cells/well in 96-well plates. After infection, cells were grown in DMEM supplied with 1% FCS. SARS-related coronavirus 2, isolate USA-WA1/2020 (BEI Resources, Cat. no. NR-52281) was used for production of the virus stock for the test.

25 mg of the photocatalytic powder (mechanical mixture of 17.5 mg of BiOCl_0.2_Br_0.8_ with 7.5 mg of BiOBr) was mixed with 1 mL of the working SARS-CoV-2 stock in sterilized glass vials and incubated under visible light (10W Daylight LED lamp^37^) and under dark conditions for indicated time intervals. After the incubation, the virus–compound mixtures were transferred into 1.5 mL of Eppendorf tube and spun down (2500 rpm, 3 min, 4 °C). The supernatants were subjected to serial dilutions in the DMEM medium without FCS, and 50 μL of the diluted virus were added to Vero-E6 cells for absorption. The test was performed in triplicates. After 1 h of incubation, 150 μL of fresh medium was added (DMEM, 1% FCS final concentration). Cells were incubated for another 48 h (CO_2_ 5%). Mock virus samples were incubated in glass vials without the photocatalytic powder. 48 h post infection, cells were fixed with 4% formaldehyde for 30 min, then washed with PBS and stained with Crystal Violet (0.05%) for 10 min. After removing the stain, the wells were analyzed for the cytopathic effect (CPE), while wells empty of cells were detected as CPE-positive and purple-stained monolayers were detected as CPE-negative. Calculation of the viruses’ titers in each sample was performed according to the modified Ramakrishnan Formula (DOI: 10.13140/2.1.47770.1209).

#### 2.2.2 Vesicular Stomatitis Virus (VSV)

HeLa cells were grown in NUNK plates, of 10 cm diameter, using DMEM (Dulbecco’s Modified Eagle’s Medium) manufactured by Beit Ha Emek, Israel, with the addition of 10% FCS (Fetal Calm Serum), glutamine, and a mix of antibiotics penicillin/streptomycin.

The infection was held in 96-well plates, 24 h after the cell’s growth. The cell cultures were infected with 50 μL of virus in decimal dilutions. After an hour of the virus’s transmission, the cultures were covered in 150 μL of DMEM with an addition of 2% FCS. The virus calibration was performed 48 h after the infection. The cultures were fixated using 1.7% formaldehyde for 30 min at room temperature. They were stained using 100 μL of 0.01% crystal violet and washed using tap water.

For the test procedure, 10 mg of the photocatalytic mixture (mechanical mixture of 7 mg of BiOCl_0.2_Br_0.8_ with 3 mg of BiOBr) was mixed with the virus in 1 mL of medium in a glass tube. The test was conducted for an hour, in room temperature, with continuous shaking, under controlled light conditions (LED lamp). Simultaneously, control test was conducted in the dark, while the tube was wrapped using aluminum foil to prevent light penetration. Virus samples were collected at intervals of 10 min. Samples were centrifuged to separate the photocatalyst (nonsoluble powder) from the virus. After the centrifuge, each sample was diluted with decimal dilutions, and 50 μL virus was transferred to the HeLa cell culture. After the cell’s infection with the virus samples, 150 μL medium was added to each well and the cells were incubated at 37 °C for 48 h. The virus calibrate was determined as the last dilution where the cytopathic effect was observed (end-point dilution). At the same time, the photocatalyst’s toxicity was tested. HeLa cells were grown as described earlier in the DMEM medium, incubated with the photocatalyst in light and dark conditions.

It is important to mention that each antiviral test was conducted three times to confirm the durability and stability of the as-synthesized composite.

### 2.3 Synthesis of the Photocatalysts

BiOCl_0.2_Br_0.8_ was synthesized by dissolving 9.2 g of bismuth nitrate in 80 mL of 1:1 deionized water and glacial acetic acid mixture, stirring for 15 min until a transparent solution was formed. Subsequently, 5.5 g of cetyltrimethylammonium bromide (CTAB) was dissolved in 25 mL ethanol and 10 mL deionized water, and 4.8 g of 25 wt % aqueous solution of cetyltrimethylammonium chloride (CTAC) was added simultaneously to the above solution and stirred for another 60 min at room temperature. The precipitate was filtered and washed a couple of times with ethanol as well as with deionized water to remove nonreactive organic species. The precipitate was then dried in air for 12 h.

BiOBr was prepared by the same synthetic process as BiOCl_0.2_Br_0.8_ without the addition of cetyltrimethylammonium chloride (CTAC).

The heterostructured composite was attained by a mechanical mixing of 70 wt % of BiOCl_0.2_Br_0.8_ with 30 wt % of BiOBr. The resulting yellowish solid is depicted in Figure S1.

### 2.4 Characterization

The X-ray powder diffraction measurements were acquired via the aid of diffractometer (D8 advance, Bruker AXS, Karlsruhe, Germany), with a goniometer radius 217.5 mm, secondary graphite monochromator, 2° Soller slits, and 0.2 mm receiving slit. Achieving XRD patterns with the range of 5° to 70° 2θ occurred via CuKα radiation (λ = 1.5418 Å) with measurement conditions: tube voltage of 40 kV, tube current of 40 mA, step-scan mode with a step size of 0.02° 2θ, and counting time of 1s per step, at room temperature. Samples of as-synthesized BiOCl_0.2_Br_0.8_, BiOBr, as well as a mechanical mixture of both were located on sample stage that is regulated along the vertical axis.

High-resolution scanning electron microscopy (HRSEM) Apreo 2 S LoVac (Thermo Fisher Scientific) supplied with UltraDry EDS detector (Thermo Fisher Scientific) assisted the chemical and morphological analyses.

Transmission electron microscopy (TEM) imaging and high-resolution scanning TEM (STEM) imaging were carried out by a Themis Z aberration-corrected scanning transmission electron microscope (Thermo Fisher Scientific) operated at 300 kV and equipped with a Ceta camera, HAADF detector for STEM.

The surface area and pore radius were determined by the N_2_ Brunauer–Emmett–Teller (BET) method (NOVA-1200e) and the Barrett–Joyner–Halenda (BJH) method, respectively.

## 3 Results and Discussion

### 3.1 Characterization

**Figure 1:**
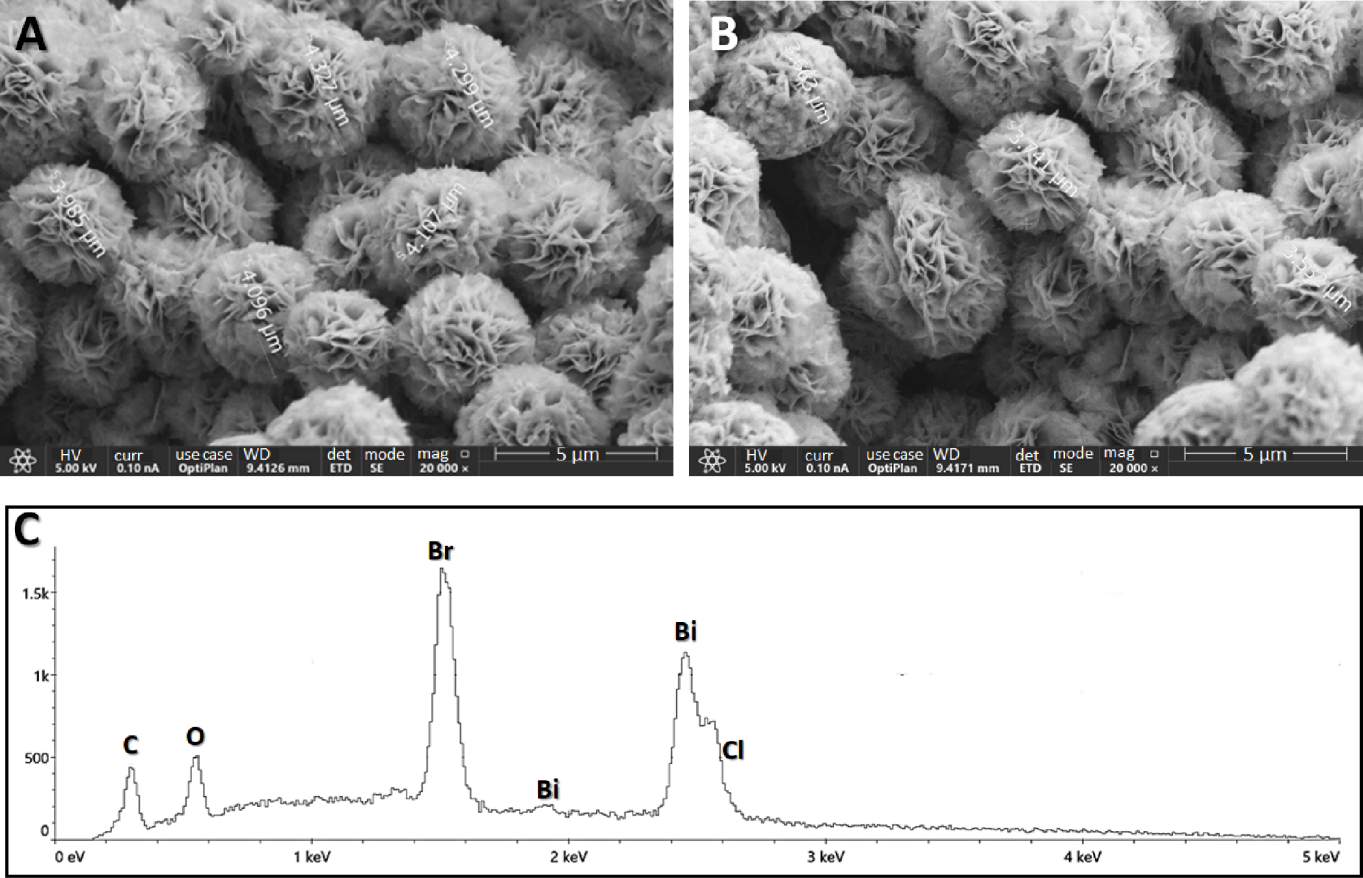
SEM images acquired from the dry powder of the mechanical mixture of as-synthesized 70%W BiOCl_0.2_Br_0.8_ and 30%W BiOBr (A and B). EDS spectrum of the same sample (C) was acquired at 5 kV accelerating voltage.

With the aid of high-resolution SEM, the morphologies and the topographical details of the mechanical mixture of dry as-synthesized (70%W BiOCl_0.2_Br_0.8_ and 30%W BiOBr) were visualized (Figure 1A,B).

The particles of the heterostructured sample are microspheres, about ∼3–4 μm in diameter, composed of thin plates with lateral dimensions of hundreds of nanometers. The plate’s thickness is about 10 nm. Consistent with the morphology of each of the photocatalysts separately, as shown in the SEM images in Figure S2A,B.

The EDS spectrum presented in Figure 1C verified the chemical composition of the material, as it consists of Bi, O, Cl, and Br. The atomic and weight percentages of the elements are shown in Table 1. Quantitative analysis of the mole ratio of Br: Cl was evaluated to be 5.96, similar to the experimental ratio of the mechanical mixture of 70%W BiOCl_0.2_Br_0.8_ + 30%W BiOBr, i.e., 6.14.

**Table 1:** EDS Atomic and Weight Percentages of the Hetero-Structured BiOCl_0.2_Br_0.8_–BiOBr at 5 kV Accelerating Voltage

element	atomic %	atomic % error	weight %	weight % error	net counts
**C**	10.0	0.2	1.1	0.0	2 768
**O**	14.7	0.6	2.1	0.1	2 276
**Cl**	5.2	1.0	1.6	0.3	642
**Br**	31.0	0.5	22.1	0.3	15 714
**Bi**	39.1	0.7	73.1	1.3	19 350

**Figure 2:**
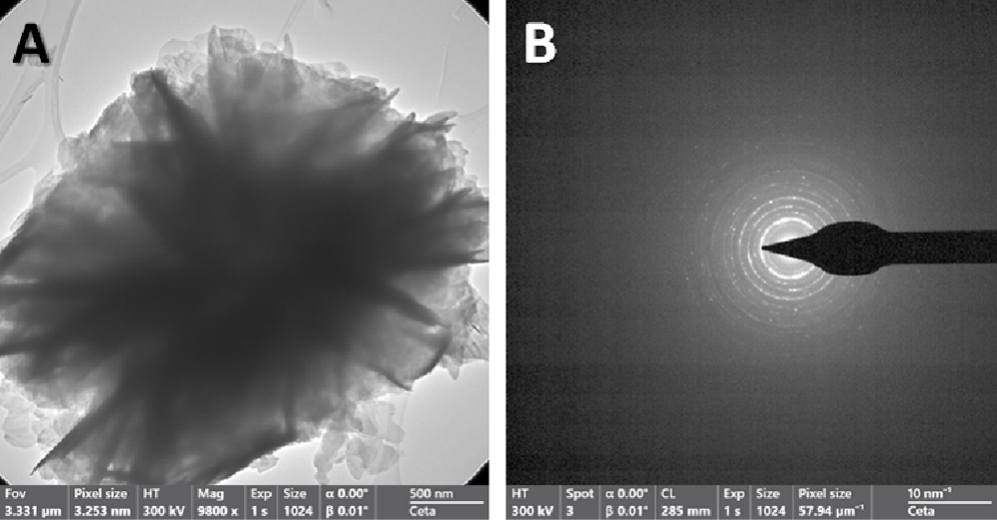
TEM image acquired from dry powder of the mechanical mixture of as-synthesized 70%W BiOCl_0.2_Br_0.8_ and 30%W BiOBr (A), and the corresponding electron diffraction pattern (B).

Corresponding to the characterization carried out by SEM, the microspheres of the heterostructured composite were revealed by TEM imaging (Figure 2A). The polycrystalline nature of the material is presented by the electron diffraction pattern (Figure 2B) of the microsphere in Figure 2A. TEM images of the microspheres of each of the photocatalysts displayed equivalent results (Figure S3).

**Figure 3:**
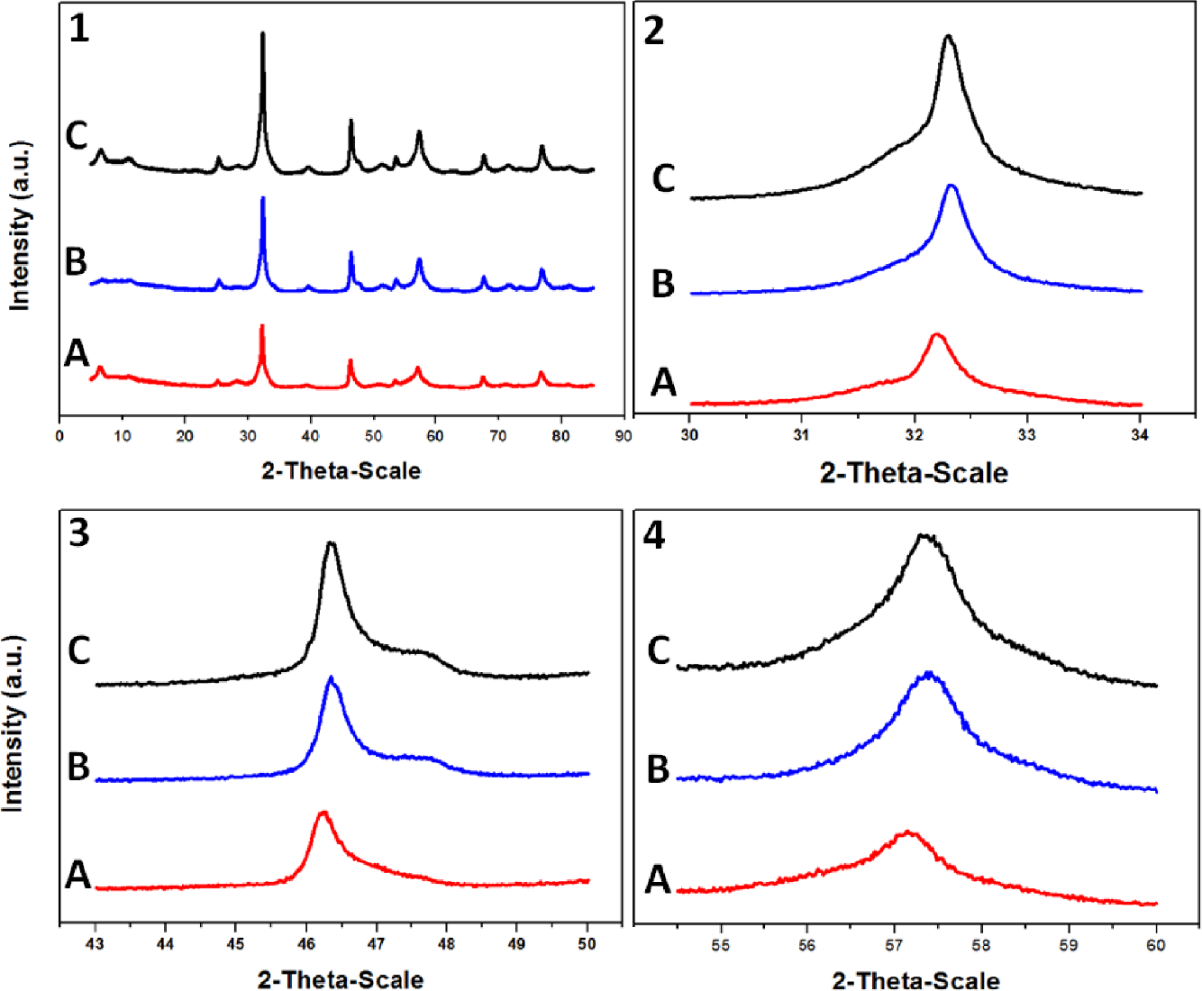
(1) XRD patterns acquired from as-synthesized BiOBr nanoparticles (A), BiOCl_0.2_Br_0.8_ (B), and the mechanical mixture of (70%W BiOCl_0.2_Br_0.8_–30%W BiOBr) (C). (2–4) represent the magnified portion of the pattern with 2θ ranging from 30 to 34°, 43–50° and 54–60°, respectively.

The crystallinity of the photocatalytic heterostructured composite was further confirmed by XRD (Figure 3). The patterns obtained from the as-synthesized BiOBr, BiOCl_0.2_Br_0.8_, and the mechanical mixture of both are displayed in Figure 3(1). Magnified portions of the patterns with 2θ ranging from 30° to 34°, 43°–50°, and 54°–60° are presented in Figure 3(2–4), respectively.

The distinct peaks and lack of impurities in each of the three samples imply a discernible sign of a high crystallinity grade.

Both of the diffraction peaks of BiOBr and BiOCl_0.2_Br_0.8_ are distinctly observable in the combined pattern, validating the Br/Cl ratio.

**Figure 4:**
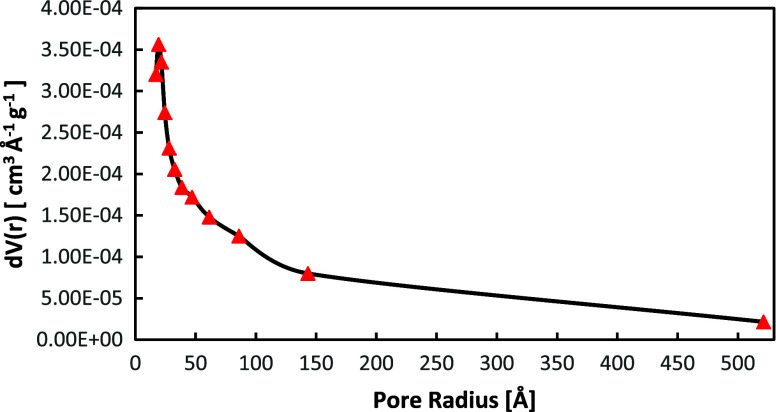
BJH pore size distribution of the mechanical mixture of 70%W BiOCl_0.2_Br_0.8_ powder and 30%W BiOBr.

The mechanical mixture of 70%W BiOCl_0.2_Br_0.8_ and 30%W BiOBr was examined via N_2_ sorption analysis and found to have a surface area of 12.052 m^2^/g and a pore radius of 19.025 Å (Figure 4). The BET isotherm of the sample is displayed in Figure S4.

### 3.2 Virucidal Activity

SARS-CoV-2 and vesicular stomatitis virus (VSV) were used as models to test the virucidal activity of the heterostructured composite of (70%W BiOCl_0.2_Br_0.8_–30%W BiOBr).

Tests were performed on the highly studied COVID-19-triggering agent, SARS-CoV-2. The yellow powder of the mechanical mixture of (70%W BiOCl_0.2_Br_0.8_ and 30%W BiOBr) was added to the working SARS-CoV-2 stock in sterilized glass vials, incubated under continuous shaking in both visible light and dark conditions for indicated time intervals. Virus–compound mixtures were centrifuged, and the supernatants were serially diluted after which they were added to Vero-E6 cells, where they typically infect them.^38^ 96-well plates were utilized for the infection process. 48 h post infection, cells were fixed with 4% formaldehyde for 30 min, then washed with PBS and stained with crystal violet (0.05%) for 10 min. After stain removal, the wells were analyzed for the cytopathic effect (CPE).

Control tests were conducted by incubating the virus samples in glass vials without photocatalytic powder.

**Figure 5:**
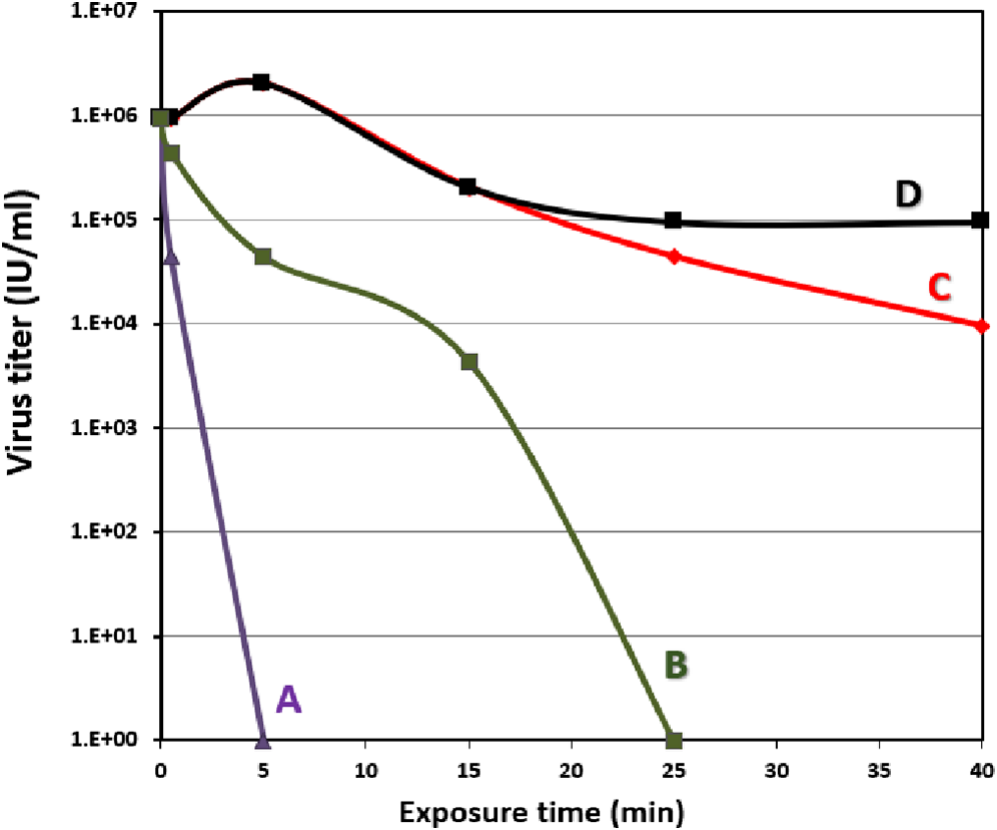
SARS-CoV-2 infectivity Assay. SARS-CoV-2 viability as a function of time over photocatalytic activity induced by the mechanical mixture of (70%W BiOCl_0.2_Br_0.8_ and 30%W BiOBr) over visible light (A) and dark light (B). (C) and (D) represent control experiments conducted over both light and dark conditions, respectively.

The SARS-CoV-2 infectivity assay is presented in Figure 5. The viability of the virus lessened drastically as a function of time after switching the LED light on and initiating the photocatalyst’s activity (from 10^6^ IU/ml to zero). Five minutes were sufficient for the photocatalytic heterostructured composite to generate ROS that unselectively breaks down the organic molecules on the viral cell wall and fully eradicates the virus (Figure 5A). An exceptionally impressive elimination rate of the COVID-19 causing virus, proving the supremacy of the studied photocatalytic mixture of 70%W BiOCl_0.2_ Br_0.8_ and 30%W BiOBr over many other photocatalysts that were found to be less effective specifically while applying harsh conditions (very intense illumination UV sources) for carrying out the inactivation of SAR-CoV-2.^39^ TiO_2_/Ti photocatalyst coating balls exhibited an antiviral activity reaching 99.99% within 6 h under UV irradiation.^40^

Cu_*x*_O/TiO_2_ photocatalyst reduced 4 orders of magnitude of SARS-CoV-2 after 2 h under regular indoor lighting.^41^

The infectivity of SARS-CoV-2 WK-521 strain decreases significantly by the photocatalytic reaction of WO_3_ for 240 min.^42^

The viral inactivation efficiency of our photocatalytic mixture surpases all the above-mentioned photocatalysts antiviral activity, as well as many more that were previously studied.^39^

Interestingly, the virus was also completely eliminated while testing the photocatalytic activity of the mechanical mixture in dark conditions (Figure 5B). Although the elimination time was 25 min, i.e., longer than the light-induced inactivation, it indicates a possible antiviral activity under dark conditions. The possible reason for the dark induced activity of the mixture of (70%W BiOCl_0.2_Br_0.8_ and 30%W BiOBr) could be impairment of the viral envelope encapsulating the RNA genome leading to growth reduction, contributed to direct contact between the nanoheterostructured composite and SARS-CoV-2. Bismuth oxyhalides were previously found to cause physical harm to microbe’s cell wall via direct contact.^43,44^ Additional motive could be related to reactive oxygen species (ROS) generation in dark conditions, by the interaction between the dissolved oxygen and the surface defects of the oxide or oxyhalide.^45^

The control tests executed under LED illumination showed a slight growth inhibition as compared to that in the dark (Figure 5C,D), indicating that SARS-CoV-2 virus is sensitive to light.

Regarding VSV, test conditions similar to those of the previously discussed SARS-CoV-2 virus elimination analysis were applied. Tests were performed in glass tubes including the photocatalytic powder mixed with the virus suspended in 1 mL growth medium (Figure S5). The tests were conducted for 1 h each, at room temperature and under sterile conditions. The glass tubes were continuously shaken to confirm samples’ homogeneity throughout the test duration. Initiation of the photocatalyst’s activity occurred after switching the LED lamp light on. Virus samples were collected at 10 min intervals after the start of each test, centrifuged to separate nonsoluble powder, i.e., the photocatalyst, from the virus and diluted with decimal dilutions. The collected samples were transferred to HeLa cell culture, as VSV is known to cause cytopathic effect on said cells.^46^ The infection was held in 96-well plates, 24 h after the cell’s growth. The cells were incubated at 37 °C for 48 h following the infection after which the cultures were fixated using 1.7% formaldehyde for 30 min at room temperature. They were stained using 100 μl 0.01% crystal violet. The virus titer was calculated via the end-point dilution assay, i.e., the last dilution where the cytopathic effect was perceived.

Simultaneously, control tests were conducted in the dark, while the tubes were wrapped by using aluminum foil to prevent light penetration.

**Figure 6:**
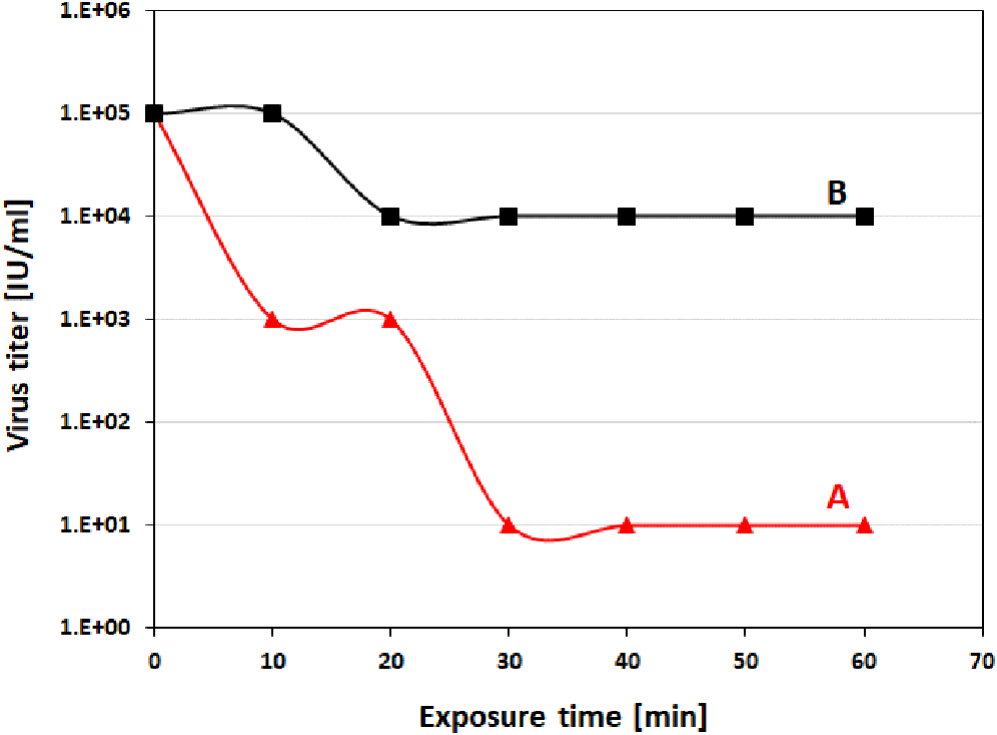
Vesicular stomatitis virus infectivity assay. VSV viability as a function of time over photocatalytic activity induced by the mechanical mixture of (70%W BiOCl_0.2_Br_0.8_ and 30%W BiOBr) over light (A) and dark (B) conditions.

The virucidal activity of the photocatalytic heterostructured composite is shown in Figure 6. Remarkable inhibition of viral growth occurred after the visible light was switched on, i.e., activating the photocatalytic mixture and generating reactive oxygen species (ROS), the oxidizing agents that handle deleterious pathogens. A reduction of four-folds transpired only after 30 min of illumination (Figure 6A). This slower operating time as compared with the previous case of SARS-CoV-2, was anticipated since we used less photocatalytic material (10 mg) against a similar starting viral titer. This was implemented to test the photocatalytic mixture behavior at lower quantities against a structured virus similar to SARS-CoV-2.

The viral viability was slightly affected in the dark conditions, where the photocatalyst was not activated via the light (Figure 6B). The same interesting behavior as demonstrated previously with SARS-CoV-2, apart from the fact that a reduction of only one-fold was achieved here owing to the lower amount of heterostructured composite used, thereby minimizing both the chances of direct contact between the photocatalyst and the virus in the tested suspension as well as the ROS generated in the dark conditions.

The control tests where the virus was suspended in the growth medium without any presence of the photocatalytic material showed no antiviral activity (Figure S6).

The previous results manifest the superior photocatalytic activity of the heterostructured composite against vesicular stomatitis virus.

The photocatalyst’s toxicity on HeLa cells showed no cytotoxic effect in light and dark conditions at the concentration chosen to eliminate the virus, i.e., 10 mg/mL (Figure S7), thus supporting the photocatalyst’s selectivity toward the virus.

### 3.3 Mechanistic Study

The technique employed by the heterostructured composite to overpower the studied model viruses was further explored.

The generated hydroxyl radicals and super oxide anion radicals can degrade the viral components, distorting its structural integrity and interfering with its biological functions.^47^

The viral inactivation process occurs by the destruction of the virus’s capsid through its reaction with the reactive oxygen species, as a result the genetic materials are released causing the deactivation process. Photocatalysis acts on surfaces, oxidizing the viral matter where the final phase ends in the formation of CO_2_ and water.^39,40^

The free radicals generated by the yellowish photocatalytic powder in the tested viral suspensions were defined as hydroxyl radicals and super oxide anion radicals. This result was realized knowing that they can both react to form hydrogen peroxide (H_2_O_2_), hence testing for H_2_O_2_ presence, as thoroughly explored in our previous study^37^ in the suspension test guarantees the stated findings.

The dry as-synthesized photocatalytic composite was mixed with deionized water, followed by visible light illumination. Hydrogen peroxide formation test strips were used a couple of minutes past the material’s activation to measure the production and concentration of H_2_O_2_ in the tested suspension, where it was ascertained to be 0.05–0.3 ppm.

## 4 Conclusions

The suggested heterostructured composite of 70%W BiOCl_0.2_Br_0.8_ and 30%W BiOBr showed exceeding elimination efficacy against stubborn enveloped viruses.

The composite’s photocatalytic activity had a significant impact on the pandemic caused by SARS-CoV-2 since it performed not only with a light source but also in its absence, intensifying the exceptionality and the significance of the developed photocatalytic system.

This composite could be of great performance in diverse applications, such as water disinfection and air treatment. It can also be beneficial in dark spaces, where it is not feasible for light to exist.

This study sheds light on the applied aspects of antiviral photocatalysis for both indoor and outdoor applications.

## Supporting Information Available

Figure S1: Photocatalytic dry powder; Figure S2: SEM images acquired from pure as-synthesized BiOCl_0.2_Br_0.8_ (A) and BiOBr (B); Figure S3: TEM images acquired from pure as-synthesized BiOCl_0.2_Br_0.8_ (A) and BiOBr (B); Figure S4: Nitrogen sorption isotherm of 70%W BiOCl_0.2_Br_0.8_ and 30%W BiOBr photocatalytic powder; Figure S5: The glass vials containing the antiviral suspension as well as the photocatalytic powder; Figure S6: VSV control experiments; Figure S7: Photocatalytic toxicity on HeLa cells (PDF)
