# Supplementary material for: Photocatalytic-Driven Antiviral Activities of Heterostructured BiOCl0.2Br0.8 – BiOBr Semiconductors
Source: ACS Omega. 2024 Apr 10;9(16):18183–90. doi: 10.1021/acsomega.3c10310 (PMC11044170; doi:10.1021/acsomega.3c10310)
Supplement: Supplementary file 1 — ao3c10310_si_001.pdf [file ao3c10310_si_001.pdf]

Photocatalytic driven antiviral activities of hetero-structured $BiOCl_{0.2}Br_{0.8}$ - BiOBr semiconductors.

Razan Abbasi, Hani Gnayem and Yoel Sasson*.*

Razan Abbasi - Casali Center of Applied Chemistry, Institute of Chemistry, The Hebrew University of Jerusalem, 9190401, Israel.

Email: Razan.abbasi@mail.huji.ac.il

Prof. Yoel Sasson - Casali Center of Applied Chemistry, Institute of Chemistry, The Hebrew University of Jerusalem, 9190401, Israel.

Email: ysasson@huji.ac.il

Dr. Hani Gnayem – Casali Center of Applied Chemistry, Institute of Chemistry, The Hebrew University of Jerusalem, 9190401, Israel.

Email: Hani.gnayem@mail.huji.ac.il

Supporting Information

Photocatalytic Powder. A digital photo of the dry powder of the mechanical mixture of as-synthesized 70%W $BiOCl_{0.2}Br_{0.8}$ and 30%W BiOBr is shown in Figure S1.

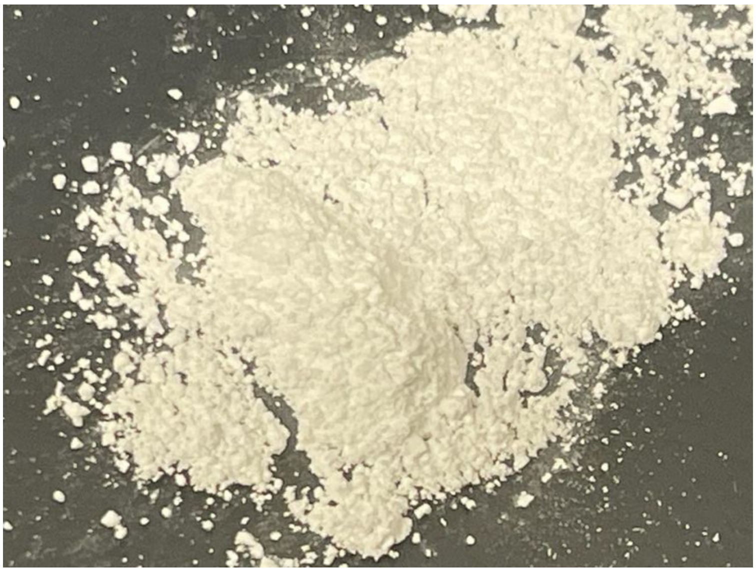

Figure S1. Photocatalytic dry powder.

SEM image. SEM images acquired from pure as-synthesized $BiOCl_{0.2}Br_{0.8}$ and BiOBr is shown in Figure S2.

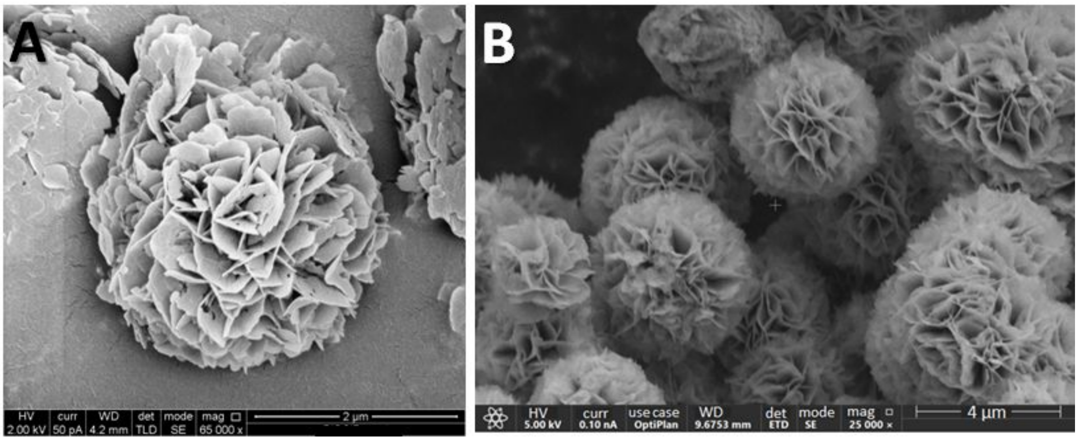

Figure S2. SEM images acquired from pure as-synthesized $BiOCl_{0.2}Br_{0.8}$ (A) and BiOBr (B).

TEM image. TEM images acquired from pure as-synthesized $BiOCl_{0.2}Br_{0.8}$ and BiOBr are shown in Figure S3.

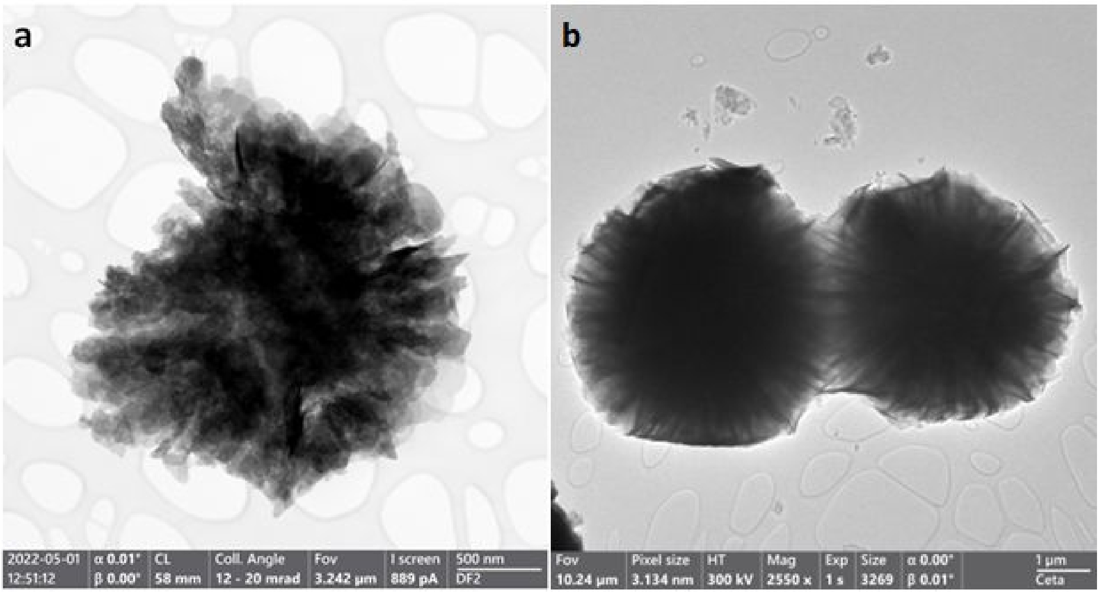

Figure S3. TEM images acquired from pure as-synthesized $BiOCl_{0.2}Br_{0.8}$ (a) and BiOBr (b).

BET surface area. Nitrogen sorption isotherm of the hetero-structured composite of 70%W $BiOCl_{0.2}Br_{0.8}$ and 30%W BiOBr powder is shown in Figure S4.

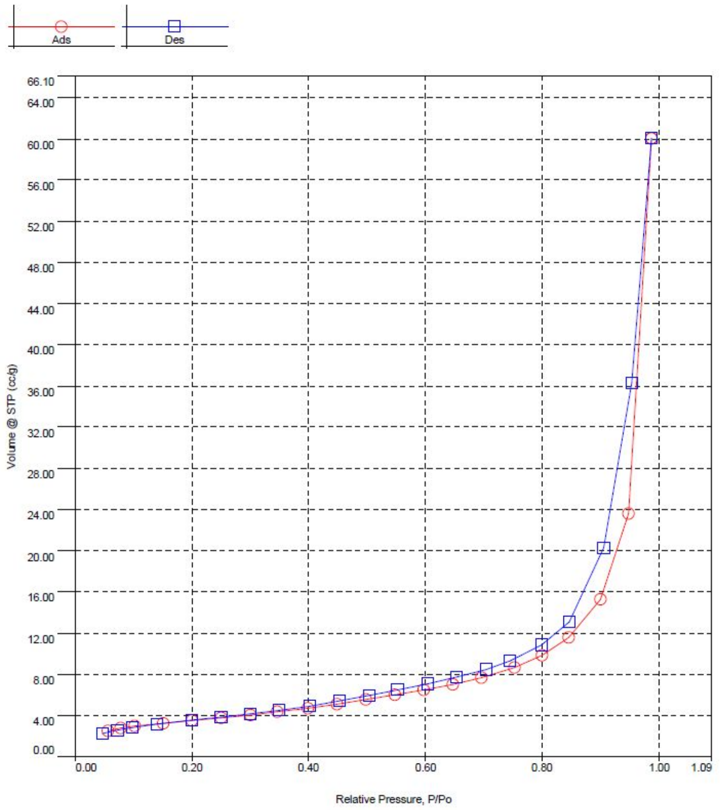

Figure S4. Nitrogen sorption isotherm of **70%W $BiOCl_{0.2}Br_{0.8}$ and 30%W BiOBr** Photocatalytic powder.

Antiviral activity test conditions. The glass vials in which the antiviral tests took place is shown in Figure S5.

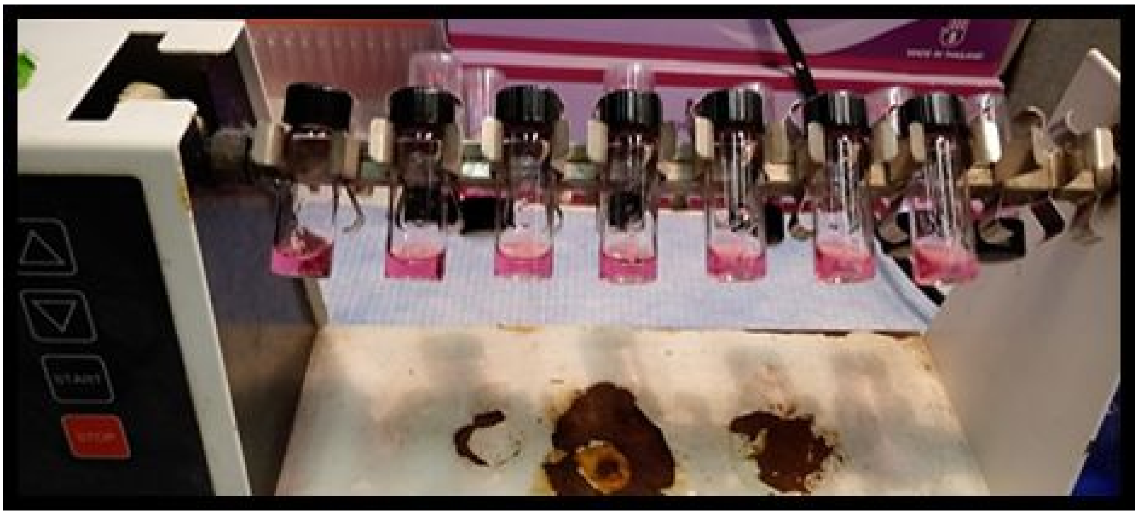

Figure S5. The glass vials containing the antiviral suspension as well as the photocatalytic powder.

VSV Antiviral activity test control experiments. The control experiments conducted over both light and dark conditions, respectively are shown in Figure S6.

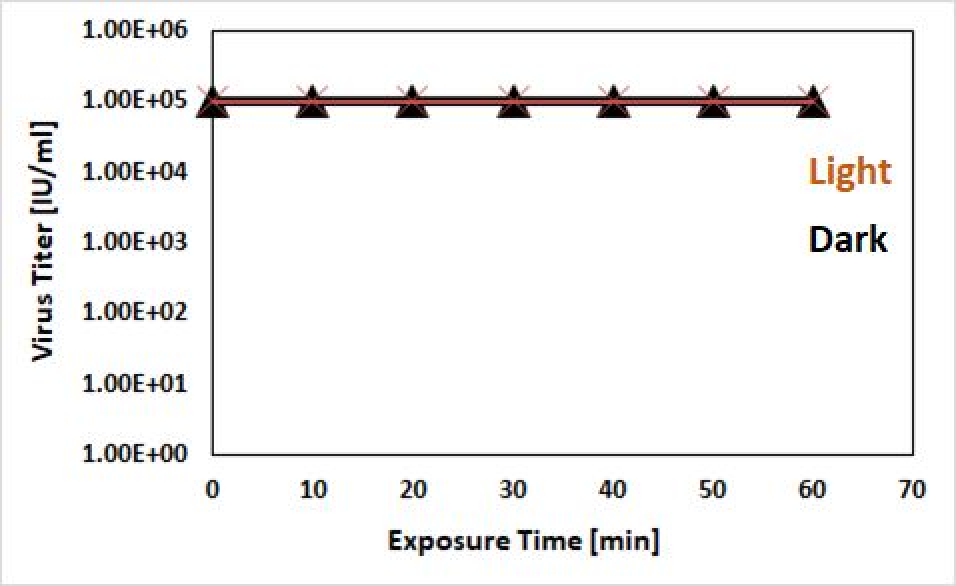

Figure S6. VSV control experiments.

Photocatalytic toxicity on HeLa cells. HeLa cells were fixed and stained with crystal violet in 96 well plate post infection. Purple-stained wells indicate no cytopathic effect, while white (i.e., empty) wells indicate cytopathic effect. Several dilutions of the virus are presented in Figure S7.

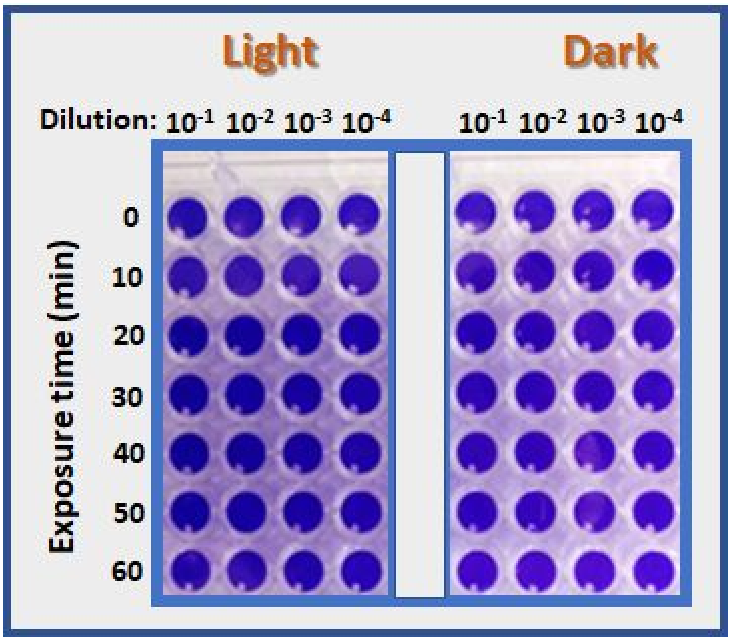

Figure S7. Photocatalytic toxicity on HeLa cells.
